# Genetic diagnosis of macrotia in PIK3CA-Related Overgrowth Spectrum (PROS) and long-term outcome of otoplasty: a case report and literature review

**DOI:** 10.1016/j.bjorl.2026.101766

**Published:** 2026-02-05

**Authors:** Lili Chen, Xuerui Hu, Jingwen Li, Chenlong Li, Jing Ma, Aijuan He, Yaoyao Fu, Tianyu Zhang

**Affiliations:** a Fudan University, Eye and ENT Hospital, Department of Facial Plastic and Reconstructive Surgery, Shanghai, China; b Fudan University, Eye and ENT Hospital, ENT Institute, Shanghai, China; c Fudan University, NHC Key Laboratory of Hearing Medicine, Shanghai, China

**Keywords:** PIK3CA mutations, Overgrowth syndrome, Facial infiltrating lipomatosis, Whole exome sequencing, Aesthetic outcome

## Abstract

**Objectives:**

PIK3CA-Related Overgrowth Spectrum (PROS) is a highly heterogeneous disease. Facial Infiltrating Lipomatosis (FIL) is a rare PROS subset, and its atypical phenotypes, such as macrotia, present diagnostic and therapeutic challenges due to limited data. This study aims to detail the diagnostic and long-term management procedures for an extremely rare case of Macrotia associated with PROS/FIL.

**Methods:**

To confirm the underlying etiology, Whole Exome Sequencing (WES) was performed, complementing routine clinical and pathological examinations. Otoplasty was used as the intervention to reduce ear size. Long-term follow-up was conducted to monitor surgical outcome, stability, and potential recurrence.

**Results:**

WES identified a PIK3CA pathogenic variant (p.H1047R). Combined with pathological findings, the patient was definitively diagnosed with PROS manifesting as the FIL phenotype. The reductive otoplasty surgery achieved significant improvement in the affected ear's morphology, reducing the deformity to a minimal, aesthetically pleasing level. Crucially, the 1-year follow-up showed remarkable stability in the near-normal ear contour, without any signs of recurrence or overgrowth. The successful aesthetic restoration significantly alleviated the patient's psychological distress.

**Conclusions:**

This case demonstrates that WES is essential for accurate molecular diagnosis of PROS/FIL in patients presenting with atypical phenotypes like Macrotia. Furthermore, otoplasty is an effective and reliable reconstructive strategy for restoring ear aesthetics in these patients, providing excellent and stable long-term results and improving patient quality of life.

**Level of evidence:**

Level 4.

## Highlights

• WES provides essential molecular confirmation for atypical PROS/FIL phenotypes such as macrotia.
• Delayed reductive otoplasty in adulthood yields durable and stable aesthetic outcomes in FIL-associated macrotia.
• Successful surgical management relieves patient anxiety and highlights the value of personalized treatment.

## Introduction

The PIK3CA-Related Overgrowth Spectrum (PROS) encompasses a collection of established and emerging clinical entities characterized by segmental or focal overgrowth with congenital or early childhood onset.^1^^,^^2^ The specific mutation sites and the embryonic developmental stages at which the mutation occurs influence the phenotype of PROS.^3^ Consequently, the affected cells and tissues vary among individual patients. Clinical Whole-Exome Sequencing (WES)^4^, ^5^, ^6^ has emerged as a powerful diagnostic tool in clinical medicine and is also beneficial for the diagnosis of patients with PROS.

It has been noted that a subset of patients with PROS exhibit clinical manifestations of Facial Infiltrating Lipomatosis (FIL).^7^, ^8^, ^9^ FIL is an extremely rare, progressive condition characterized by the diffuse infiltration of mature adipose tissue into facial bones and surrounding structures, with fewer than 100 cases currently reported.^10^ FIL can significantly impair both facial appearance and function. Currently, surgical intervention remains the primary treatment approach for FIL.^11^ Laser is also used as an effective tool for diminishing the volume of the FIL lesions,^12^^,^^13^ and emerging targeted therapies could present an additional treatment avenue in the future.^14^

In this study, we present the diagnosis and treatment process of a 22-year-old male patient with left-sided macrotia, an exceptionally rare phenotype associated with PROS. Whole Exome Sequencing (WES) identified a pathogenic variant in the PIK3CA gene. This finding, combined with pathological examination, led to the final diagnosis of PROS presenting as the FIL phenotype. To address the macrotia, the patient underwent otoplasty surgery, which yielded a notable, long-term stable enhancement of the ear's appearance. This study contributes valuable data to the existing literature on PROS and highlights the successful application of otoplasty as an effective long-term treatment option for macrotia in PROS.

## Methods

### Clinical data collection

Family and medical history, signs and symptoms, imaging findings, pathological results, and the complete treatment course were retrospectively reviewed from the patient's clinical records. This study was conducted in compliance with the Declaration of Helsinki and had been approved by the Institutional Review Board (nº 2020526).

### Genetic analysis and consent

Whole-Exome Sequencing (WES) was performed for the patient and both parents. Written informed consent for the study and publication was obtained from the patient. Consent for parental DNA sample contribution was obtained from both parents after the family met with a qualified geneticist and genetic counselor to ensure comprehensive understanding of the procedure and its implications.

### Clinical examinations and pathological analysis

Computed Tomography (CT) was performed to evaluate craniofacial abnormalities. Hearing status was assessed using otoscopic examination and pure-tone audiometry. For pathological analysis, surgical specimens were routinely fixed in formalin, processed, embedded in paraffin, and serially sectioned. Hematoxylin-Eosin (HE) staining was subsequently performed.

### Genetic sample processing and validation

Genomic DNA was extracted from peripheral blood using the Puregene Blood Core Kit (QIAGEN). Whole-Exome Sequencing (WES) was then performed. To isolate the somatic mutation, fibroblasts were obtained from skin explant cultures derived from the macrotia lesion. DNA was extracted from these fibroblasts using the DNA Extraction Kit (TIANGEN). We subsequently performed Sanger sequencing on this fibroblast-derived DNA to validate the PIK3CA mutation.

## Results

### Presurgical clinical characteristics of patient

**Fig. 1:**
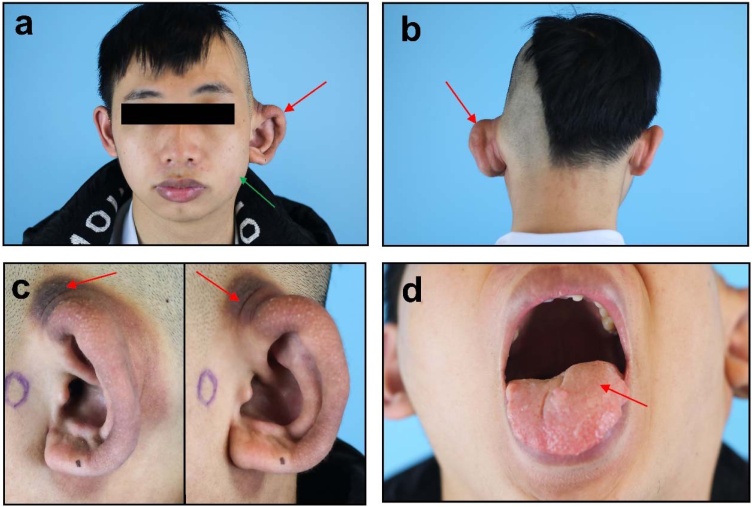
Clinical photos before treatment. (a) Front view: left-sided macrotia (red arrow) and mild hemifacial hypertrophy (green arrow). (b) Back view: left-sided macrotia (red arrow); (c) Lateral view: skin hyperpigmentation around the left ear (red arrow). (d) Mild hemi-macroglossia (red arrow).

**Fig. 2:**
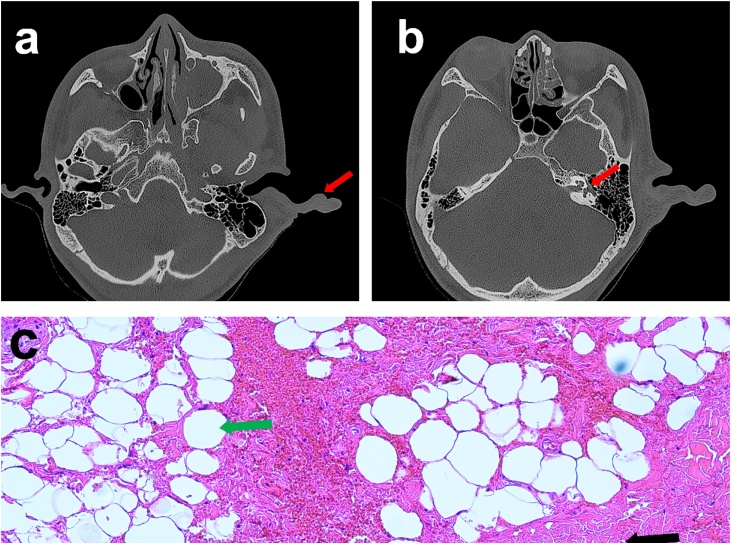
Clinical examinations. (a) CT image demonstrated left-sided macrotia deformity with mixed fat density (red arrow). (b) The picture showed the enlarged left facial nerve canal (red arrow). (c) The pathological examination (Hematoxylin-Eosin stain) indicated mature adipose tissue (green arrow) infiltrating or surrounding subcutaneous fibroconnective tissue (black arrow).

A 22-year-old man presented with left-sided macrotia, accompanied by mild hemi-macroglossia and hemifacial hypertrophy. Skin hyperpigmentation around the left ear was also observed (Fig. 1). Ear asymmetry was noted since birth and progressively worsened with growth, though there was no family history of facial abnormalities. Computed Tomography (CT) performed preoperatively revealed left-sided macrotia deformity and demonstrated mixed fat density within the adjacent soft tissues. Additionally, the left facial nerve canal was wider (Fig. 2). No fatty infiltration was observed in the osseous structures. Ultrasound examination of the hypertrophic facial area excluded the presence of a vascular malformation. An abdominal ultrasound performed to check for organomegaly was unremarkable, and pure-tone audiometry indicated normal hearing. The patient did not exhibit any common PROS associations, including epidermal nevus, hand/foot anomalies, spinal malformations, brain anomalies, or other vascular malformations. Although mild macroglossia was present, it did not significantly impact the patient's eating or speech functions.

### Genetic and pathological findings

**Fig. 3:**
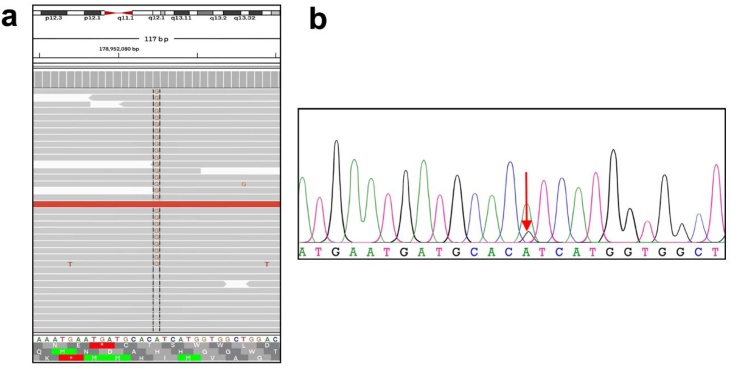
Genetic testing. (a) PIK3CA variant, c.3140A>G (p.H1047R), was identified by WES analysis, as shown on the integrated genomics viewer. (b) Sanger sequencing confirmed the mutation (red arrow).

To establish the diagnosis, a partial biopsy of the subcutaneous tissue in the macrotia was obtained. DNA was extracted from the patient's and parents' peripheral blood samples for WES analysis. The biopsied subcutaneous tissue was also processed for pathological examination via routine embedding and slicing. The pathological examination exhibited cohesive accumulations of adipose connective tissue, accompanied by localized fibrous tissue hyperplasia and degeneration (Fig. 2c). Additionally, a small number of glands, nerves, and minor blood vessels were sporadically observed. Due to the unusual nature of the infiltration, a definitive diagnosis could not be established by the pathologist, who suggested a highly rare syndrome. WES analysis subsequently revealed the presence of a pathogenic point mutation c.3140A>G (p.H1047R), in the PIK3CA gene. The mutation was detected solely in the patient's macrotia tissue sample, and was confirmed to be a somatic mosaic with a variant allele frequency of 15.1%, verified by Sanger sequencing (Fig. 3). This variant was not detected in the blood samples. Based on the synthesis of physical examination, genetic testing, and pathological examination, the patient was diagnosed with PROS (PIK3CA-related overgrowth spectrum) presenting with the FIL phenotype.

**Fig. 4:**
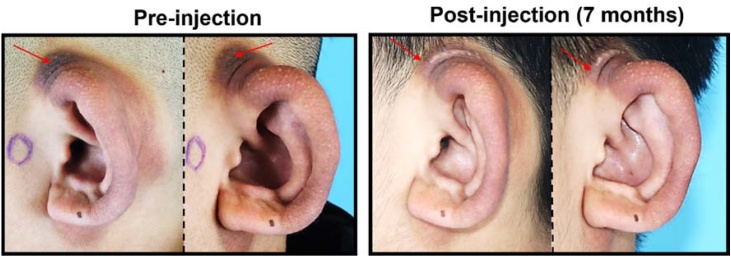
Clinical photos after bleomycin injection.

Bleomycin injection was used as a supplementary treatment due to the presence of hyperpigmentation on the affected auricle. Following the initial biopsy, bleomycin was injected subcutaneously into the affected tissue. Upon follow-up seven months after the injection, a clear reduction in the hyperpigmentation was observed (Fig. 4).

### Aesthetic restoration of macrotia and long-term outcome

Given that the macrotia deformity was the most noticeable manifestation and had the greatest impact on the patient's appearance, surgical intervention was prioritized to reduce the macrotia size and improve the aesthetic outcome. The patient underwent reductive otoplasty seven months after the initial biopsy.

**Fig. 5:**
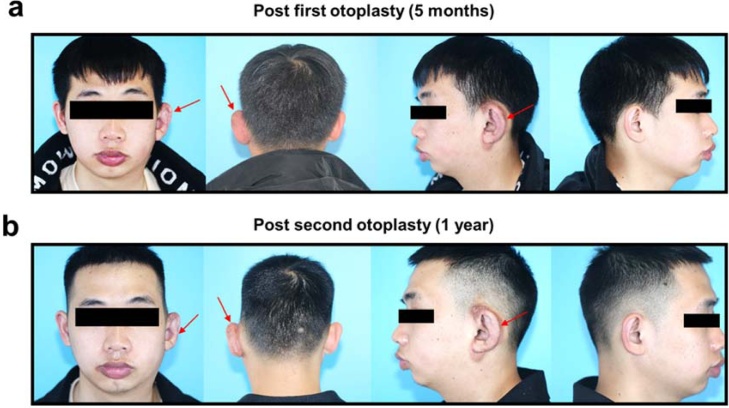
Clinical photos after reductive otoplasty. (a) Five months after the first otoplasty. (b) One year after the second otoplasty.

Postoperatively, a reduction in the auricular size and a decrease in thickness were observed (Fig. 5a). To achieve a more aesthetically pleasing appearance, the patient required a minor refinement otoplasty five months after the initial procedure. The final morphology of the affected ear significantly improved following the second surgery, resulting in few noticeable deformities. The patient reported an enhanced quality of life and significant improvement in mood. The final follow-up visit took place one year after the second otoplasty, demonstrating excellent maintenance of the aesthetic outcome without any evidence of macrotia regrowth (Fig. 5b).

## Discussion

PROS (PIK3CA-related Overgrowth Spectrum) proposed in 2015, represents a collection of rare genetic disorders characterized by asymmetrical overgrowth. This spectrum results from somatic gain-of-function mutations in the PIK3CA gene, which encodes the phosphatidylinositol 4,5-bisphosphate 3-Kinase Catalytic subunit alpha (PI3K-alpha).^15^ PI3K-alpha protein plays a pivotal role in the regulation of cell growth, proliferation, and survival via the PI3K/AKT/mTOR pathway. Facial Infiltrating Lipomatosis (FIL), a rare congenital condition with fewer than 100 cases reported in the literature, is distinguished by widespread adipose infiltration within the soft tissues of the face. Based on increasing clinical and sequencing evidence, a clear association between PIK3CA gene mutation and FIL has been established.^7^^,^^9^ Consequently, FIL is now categorized as a subset within the PROS spectrum.

Several possible explanations exist for how the somatic PIK3CA mutation drives the FIL phenotype. First, the phenotype is likely influenced by the timing of the somatic mutation occurrence during embryogenesis. An early genetic mutation may correlate with the origin of the organs commonly impacted by FIL, which are derived from the mesenchyme of the first and second branchial arches.^16^ A second explanation involves the specific cellular lineage carrying the mutation. The emergence of FIL could potentially be attributed to a mutation arising in craniofacial neural crest cell.^17^ This lineage is critical, as it gives rise to numerous structures relevant to FIL, including the ear, maxilla, and mandible.

The variable phenotype spectrum of PROS/FIL necessitates molecular confirmation, as accurate diagnosis cannot be established based solely on clinical features. Molecular analysis, such as targeted sequencing or genotyping for PIK3CA mutations, is essential to confirm the diagnosis and differentiate this condition from similar disorders.^4^^,^^9^^,^^18^ This highlights the clinical utility of genetic testing in facilitating the accurate and timely diagnosis of PROS/FIL.

The association of the PIK3CA gene mutation with the disease also opens up possibilities for targeted therapies intervention. PI3K inhibitors have been developed and tested in clinical trials for various conditions driven by PIK3CA mutations.^19^^,^^20^ Specifically in the context of FIL, targeting the aberrant PI3K pathway activation may offer potential therapeutic benefits for patients.^21^ For example, the PI3K inhibitor alpelisib has been reported as an effective treatment for PIK3CA-associated FIL.^20^

The clinical features of PROS and its subset, FIL, are highly heterogeneous, making accurate diagnosis challenging, especially when patients present with atypical phenotypes like macrotia as the primary manifestation. To provide strong diagnostic justification, we employed Whole Exome Sequencing (WES), which definitively identified the PIK3CA pathogenic variant (p.H1047R). This molecular confirmation was crucial for establishing that the macrotia was, in fact, an expression of the underlying PROS/FIL and guiding our subsequent management strategy.

Furthermore, the available literature, including studies on PI3K inhibitors like alpelisib, indicates a limited pharmacological responsiveness for structural deformities like macrotia. Specifically, reports show that while systemic therapy may reduce soft-tissue thickness (e.g., lip and cheek), macrotia did not decrease in size due to the abundant extracellular matrix and limited cellularity of cartilage, thus justifying our primary focus on surgical intervention for aesthetic and functional correction.^20^

Surgical intervention remains an effective and necessary approach in the treatment of macrotia associated with FIL. Our findings align with previous reports showing successful outcomes of reductive otoplasty for this specific condition.^22^ However, the optimal timing of surgical excision for FIL remains controversial, as it directly impacts the risk of recurrence. It was reported that the post-resection recurrence rate for FIL can be as high as 62.5%,^23^ particularly in childhood and adolescence, where growth factors, such as growth hormone, may drive recurrence. Some researchers suggest that, except the rapidly progressing cases, delaying resection until adulthood could achieve stable outcomes. They believe this strategy would not only lower the overall rate of recurrence but also minimize the risk of facial nerve injury.^14^^,^^23^^,^^24^

Our study provides two critical comparative advancements: the demonstrated long-term stability of the reductive otoplasty and the validation of this delayed surgical timing. The patient in our study underwent surgery at the age of 22, ultimately achieving a durable and stable aesthetic improvement. The integrated treatment strategy, which combined preliminary injection therapy with subsequent reductive otoplasty, displayed excellent maintenance of the aesthetic outcome, with no evidence of macrotia regrowth at one-year follow-up. The aesthetic restoration also led to substantial relief from the patient's anxiety and a marked improvement in self-confidence. This successfully executed timing strategy strongly supports the perspective that delayed surgical intervention is essential for achieving stable long-term outcomes, which is a major clinical concern in managing FIL patients.

Reporting rare phenotypes is crucial for advancing clinical understanding of a disease, which directly enables more accurate diagnosis and timely treatment for affected patients. Since many PROS/FIL phenotypes have been described only in single individuals or small patient cohorts, describing additional patients is essential to fully delineate the spectrum and improve diagnostic criteria.

By highlighting the challenges inherent in the diagnosis and treatment of PROS/FIL patients presenting primarily with macrotia, this study offers valuable guidance to healthcare professionals. Clinicians can refine treatment strategies by prioritizing patient satisfaction and stable long-term aesthetic outcomes. Furthermore, this study emphasizes the importance of respecting patient preferences and the critical need for continuous, long-term patient follow-up.

The limitations of this study include the single patient focus and the relatively short follow-up time. Further studies with larger patient cohorts and longer follow-up periods are needed to assess disease progression and treatment outcomes more accurately. Studies aimed at identifying the underlying molecular mechanisms involved in the development and progression of FIL will be critical for developing novel therapeutic strategies. Finally, multi-disciplinary collaborations and the establishment of comprehensive patient registries are essential to advance our understanding of this rare and complex disease.

## Conclusion

This study provides valuable clinical experience and insights into the management of macrotia in PROS/FIL patients, and underscores the necessity of personalized treatment approaches.

## ORCID ID

Lili Chen: 0000-0001-6746-9119

Jingwen Li: 0009-0007-0146-4762

Chenlong Li: 0000-0003-0460-7937

Jing Ma: 0000-0001-6286-8153

Aijuan He: 0000-0001-9111-2654

Yaoyao Fu: 0000-0002-3197-9691

## Funding

Supported by 10.13039/501100001809National Natural Science Foundation of China (82371173, 82172105), 10.13039/100007219Natural Science Foundation of Shanghai (21DZ2200700, 20ZR1409900), and Double Excellent Foundation of Eye & ENT Hospital (SYA202003).

## Data availability statement

The data that support the findings of this study are available from the corresponding author upon reasonable request.

## Declaration of competing interest

The authors have no financial interest to declare in relation to the content of this article.
